# An automated method for identifying an independent component analysis-based language-related resting-state network in brain tumor subjects for surgical planning

**DOI:** 10.1038/s41598-017-14248-5

**Published:** 2017-10-23

**Authors:** Junfeng Lu, Han Zhang, N. U. Farrukh Hameed, Jie Zhang, Shiwen Yuan, Tianming Qiu, Dinggang Shen, Jinsong Wu

**Affiliations:** 1 Department of Neurosurgery, Huashan Hospital, Fudan University, Shanghai, China; 2 Department of Radiology and Biomedical Research Imaging Center (BRIC), University of North Carolina at Chapel Hill, Chapel Hill, NC USA; 3 Department of Brain and Cognitive Engineering, Korea University, Seoul, 02841 Republic of Korea

## Abstract

As a noninvasive and “task-free” technique, resting-state functional magnetic resonance imaging (rs-fMRI) has been gradually applied to pre-surgical functional mapping. Independent component analysis (ICA)-based mapping has shown advantage, as no *a priori* information is required. We developed an automated method for identifying language network in brain tumor subjects using ICA on rs-fMRI. In addition to standard processing strategies, we applied a discriminability-index-based component identification algorithm to identify language networks in three different groups. The results from the training group were validated in an independent group of healthy human subjects. For the testing group, ICA and seed-based correlation were separately computed and the detected language networks were assessed by intra-operative stimulation mapping to verify reliability of application in the clinical setting. Individualized language network mapping could be automatically achieved for all subjects from the two healthy groups except one (19/20, success rate = 95.0%). In the testing group (brain tumor patients), the sensitivity of the language mapping result was 60.9%, which increased to 87.0% (superior to that of conventional seed-based correlation [47.8%]) after extending to a radius of 1 cm. We established an automatic and practical component identification method for rs-fMRI-based pre-surgical mapping and successfully applied it to brain tumor patients.

## Introduction

In functional surgical neuro-oncology, the precise localization of brain language areas contributes to both maximum resection and minimum functional injury, as well as prolonging survival time and improving quality of life^1,2^. Functional imaging-based pre-surgical mapping provides essential information for intra-operative localization of eloquent regions^3,4^. Although task-based functional magnetic resonance imaging (fMRI) has been widely applied for preoperative language mapping^5–7^, there still remain concerns of instability and variability^8^. Both sensitivity and specificity were unsatisfactory according to a systematic review^9^ and a recent study^10^ using 3 T fMRI reported that the sensitivity was only 37.1%, suggesting that task-based fMRI is not yet ready for prime-time guidance of glioma resection^11^. Moreover, it is inconvenient for both doctors (difficult-to-implement and complicated experimental design) and patients (time-consuming and highly demanding)^9,12^. For patients with language deficits, such as aphasia and alexia, or cognitive deficits, who need extensive preoperative planning, poor task performance may cause failure in language mapping.

Significant evidence has suggested that spontaneous brain activity observed in resting-state fMRI (rs-fMRI) without any explicit task performance can reveal various primary and high-level cognitive systems including the language network^13–16^. Brain areas belonging to the same functional system or network share similar spontaneous blood oxygenation level-dependent fluctuations (that is, functional connectivity, or FC), which differ from those of other systems^17,18^. Over the past decade, although most researches^13,19,20^ focused on group-level rather than individual level studies, these studies did not indicate the potential of implementing rs-fMRI in individualized language network mapping for preoperative planning. In our previous study^21^, our group demonstrated the feasibility of rs-fMRI in language mapping in glioma patients using a seed-based correlation approach, indicating the promising clinical prospect of this technique.

However, in seed-based FC, the position of a seed region could greatly affect the resulting pattern of the functional system, such as the language network. Also, the Pearson’s correlation implemented in the previous study suggests sensitivity to systematic noise such as head motion and physiological nuisance signals, easily leading to false positive (mistakenly identifying non-language areas) and false negative (missing detection of putative language areas) results. Therefore, these issues have limited the clinical application of seed-based rs-fMRI in language mapping. Independent component analysis (ICA) is one of the two most commonly adopted analytical methods for rs-fMRI data^13,14^. It is a blind source separation approach without any pre-defined seed region. Tie *et al*.^22^ have tried to implement ICA to define language areas in individual healthy subjects. They successfully mapped the language networks for all 18 subjects. However, they analyzed only healthy subjects rather than tumor patients. The clinical application of ICA on individual rs-fMRI from patients remains unknown. The lesioned brain, particularly due to the mass effect and functional reorganizations caused by the tumors, may add more complexity to ICA-based rs-fMRI language mapping^23–25^. Moreover, their semi-automated data processing method requires a further visual identification step, which is not easily applicable to clinical practice because the identification of the “correct” language network-related component from the automatically suggested multiple language network candidates by doctors poses additional difficulty. Finally, in the clinical setting, the signal-to-noise ratio of rs-fMRI data could be lower than that in a research dedicated environment.

In this study, we investigated the feasibility of applying individual ICA on rs-fMRI to map language areas preoperatively in patients with brain tumors. We aimed to investigate and establish a clinically feasible and acceptable procedure for clinicians. We adopted an automated language-component identification approach, which is different from, and also easier than, the previous method^22^ and it even requires no expertise on ICA-derived components. Most importantly, the feasibility and robustness of our approach have been verified in healthy subjects from different centers as well as in tumor patients using gold standard putative language mapping results.

## Methods

We will first describe the automatic language network mapping algorithm based on individual ICA and rs-fMRI in a cohort of healthy subjects (training group, TR). Task fMRI results from the same cohort will be used for comparison with the rs-fMRI-based mapping results. Second, this method will be validated using an independent cohort of healthy subjects with a different imaging protocol (validation group, VA). Third, the language network will be mapped preoperatively in a patient group with gliomas (testing group, TE) to assess feasibility in a clinical setting. The results will finally be compared with intra-operative electrocortical mapping result which is often regarded as the gold standard for language mapping.

### ICA-based automatic language network mapping in TR group

#### Subjects

Ten healthy subjects, of ages between 24 and 54 years (with an average age of 30.9 years), including six males and four females, were enrolled in the TR group from Huashan Hospital, Fudan University. All subjects were right-handed and native Chinese speakers. In addition, none of the subjects had any previous history of neuropsychological diseases or any form of language, auditory, or visual impairments. The Huashan Institutional Review Board approved this study, and all subjects signed the informed consent form before the scan. The procedure was carried out in accordance with approved guidelines.

#### Protocol

The subjects were asked to perform a language task of picture naming. The task paradigm was a block design, with alternating “rest – task – rest – task…” blocks. Each block lasted 24 s, and equal time was allocated to task and rest periods. The task paradigm included five task blocks separated by six resting blocks. An 8 s dummy scan was performed to stabilize the magnetic field prior to MRI scanning. There were 136 time points during the entire task sessions, with a total duration of 4 min and 32 s. The pictures were projected onto a screen placed at the heads of the subjects through an MRI-compatible visual and auditory stimulation device. The subjects were required to “silently” name the pictures: this was a language task in which subjects were required to not overtly speak out the names of the presented pictures, but to covertly (silently) name them in order to avoid lip and tongue movement. During the resting state sessions, they were asked to relax and look at the “+” sign at the center of the screen (fixation).

#### Image acquisition

MRI was acquired using a 3.0 T MRI (MAGNETOM Verio 3.0 T, Siemens AG, Erlangen, Germany) with an eight-channel coil. The parameters of rs-fMRI using echo planar imaging (EPI) were set as follows: TR/TE = 2,000/30 ms, FA = 90°, slice number = 33, matrix size = 64 × 64, FOV = 220 × 220 mm, slice thickness = 3 mm, gap = 1 mm (voxel size 3.4 × 3.4 × 4 mm^3^), dummy scan = 6 s, number of acquisitions = 240. Task-based BOLD-fMRI imaging used the same parameters (but with fewer [136] acquisitions). Three-dimensional (3D) T1-weighted magnetization-prepared rapid-gradient echo (MPRAGE) imaging was applied to acquire structural images (acquired through the sagittal plane, TR/TE = 2,530/3.45 ms, FA = 7°, slice number = 176, matrix size = 256 × 256, FOV = 256 × 256 mm, slice thickness = 1 mm).

#### Language task activation analysis (group-level)

Similar to previous studies^22^, a language area template is required to automatically select the best match from the resting-state components. Using both task-related and resting-state fMRI data, the TR group was used to generate language templates. We applied Statistical Parametric Mapping (SPM8, http://www.fil.ion.ucl.ac.uk) for preprocessing and statistical analyses to generate the activation images with the conventional processing pipeline. Data at the first four time points were discarded prior to preprocessing. After slice timing, the EPI data were corrected for head motion using rigid body transformation with 6 parameters. Data with head motion exceeding 2 mm in transformation or 2° in rotation were treated as excessive head motion and should be excluded. However, no data in this group met this exclusion criteria. Following this, the data were spatially normalized to Montreal Neurological Institute (MNI) standard space, and Gaussian smoothening with an 8-mm full-width-half-magnitude (FWHM) kernel was then performed to spatially smoothen the fMRI data.

**Figure 1:**
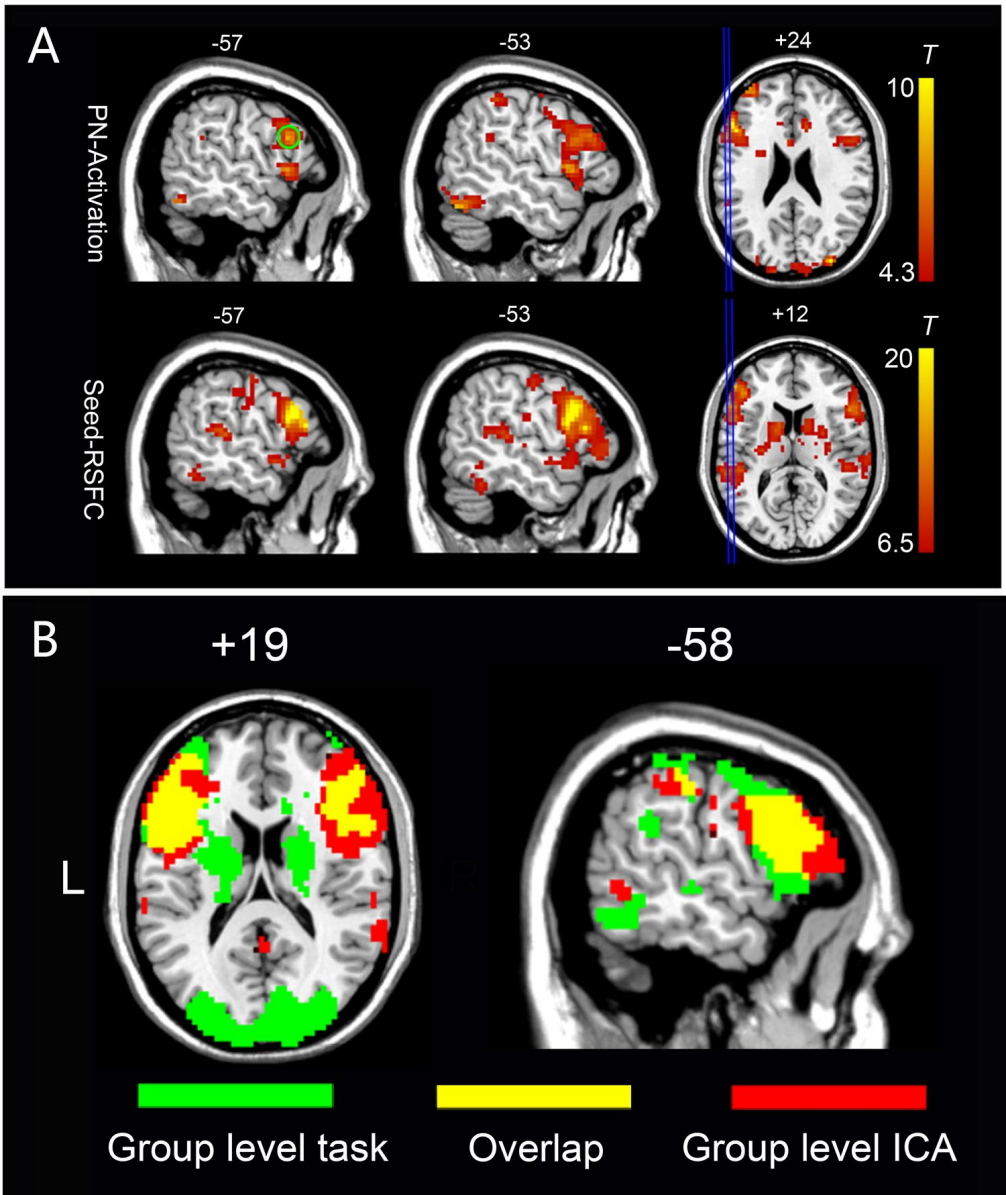
The creation of language network template and comparison of ICA and task fMRI results in the TR group. (**A**) Top: group-level task activation map in the picture naming task from the 10 subjects in the TR group (*p* < 0.005, FDR corrected); The green circle indicates the seed region [−57, 15, 24]. Bottom: group-level resting-state functional connectivity based on the seed region (*p* < 0.001, FDR corrected, extension threshold = 20 voxels), which served as template for further component identification. (**B**) Comparison between the ICA results when DICI value reached its highest value (the IC was 60) (*t* > 2.8) and the task fMRI results of the TR group (*p* < 0.005, FDR corrected; *t* > 4.3).

For task activation analysis, a generalized linear model (GLM) was used to model the individual-level task effect for each subject; following this, a random-effect model with one-sample *t*-test was applied to the individual-level contrast images from the 10 subjects to generate a group-level *t* map (language task activations). The threshold was set at *q* < 0.005 (false discovery rate [FDR] corrected) and the extension threshold was set at 20 voxels to ensure robust activation of picture naming (Fig. 1). Theoretically, the task activation map should be used as the template to identify resting-state language network; however, in practice, the group-level task activations in the picture naming task also included other task-related clusters beyond the essential language areas. Specifically, we found that the task activations also involved other functional systems such as primary and high-order visual areas (see Result), which could be due to visual stimulation during the task rather than language function. Therefore, we did not directly use the task activation as the template to avoid bias in following language network detection. Instead, we used the rs-fMRI data to generate the language template based on the group-level task activation information.

#### Creating language network template (group-level)

The rs-fMRI data was preprocessed using DPARSFA (www.restfmri.net/forum) software, which involved removing the first 10 time points, slice-timing, head motion correction, registration to an EPI standard template in the MNI space, resampling to 3 × 3 × 3 mm^3^, Gaussian smoothening (FWHM = 8 mm), temporal detrending, and band-pass filtering (0.01-0.08 Hz). Three peak coordinates were located in the frontal lobe from the group-level picture naming task activations and we attempted using all three to construct the optimal language template. The first seed [−36, 0, 51] was located in Brodmann area (BA) #6 (middle frontal gyrus), the second [−51, 27, 24] in BA #45, and the third [−57, 15, 24] in BA #44. We went on to separately use the three coordinates as seeds to generate seed-based FC maps based on the TR group. We found that the third seed led to a resting-state language FC pattern best matching with the language meta-analysis result (generated by using the keyword “language” on http://neurosynth.org, a website-based meta-analysis toolbox). This seed was then chosen as the optimal language seed region. Seed-based correlation was conducted for each subject, and the resulting Pearson’s correlation maps were transformed to *z* maps. Group-level analysis was conducted on the *z* maps using a one sample *t* test (*q* < 0.001, FDR corrected, extension threshold = 20 voxels), producing a binarized template of the language networks for automatic component identification. We named this template the “TR template” as it was derived from the TR group.

#### Group ICA analysis (data quality validation)

Group-level ICA (GICA) was first performed to validate our data quality by comparing the resultant group level intrinsic connectivity networks, including the language network, with previous data^18,22,26,27^. Individual ICA was then performed for each subject. To perform GICA, a temporal concatenation GICA (SOI-GICA, implemented in MICA toolbox, https://www.nitrc.org/projects/cogicat/) was used. The preprocessed data without detrending and filtering was fed into MICA. GICA was calculated 100 times with the initial value and the subject order randomized each time to obtain consistent result^28^. As model order (i.e., total number of components, TNC) is difficult to predefine and can significantly affect ICA results^29,30^, we deliberately set various model orders ranging from 20 to 60, with 10 in each increment, to determine the optimized model order (i.e., total component number) for the language network.

#### Individual ICA analysis (language network detection)

Individual ICA was also conducted using the MICA toolbox with a procedure similar to that in GICA, except that data from only one subject was used each time (there was no subject order randomization). Another difference is that individual ICA was applied to fMRI data in individual space, rather than the standard MNI space. Therefore, un-registered data was fed into individual ICA, while the normalization step was only done to obtain the deformation information through which the language network template in the standard space could be projected back to the individual-specific space (i.e., native space).

#### DICI algorithm

To automatically identify the language network from individual ICA results, we utilized a template matching algorithm, namely Discriminability Index-based Component Identification (DICI), which includes two modules. The first module is DICI calculation using discriminability index (also known as d-prime or d’) as a metric which compares the similarity between each component and the template. This is a useful concept in the Signal Detection Theory, which can be described using a decision-making scenario. In a simple forced choice problem, the experimenter has to correctly identify the existence of signal from a noisy background. The decision-making takes place in the presence of uncertainty caused by either external noise (controllable) or internal noise (uncontrollable and immeasurable, reflecting the variable internal response of “judgment-related neurons” in the experimenter’s brain). The higher both types of noise levels tend to be, the lower the detectability becomes. To conclude, DICI provides a precise language and graphical notation for analyzing decision making by measuring the signal strength, that is, to what extent the two distributions overlap with each other with the ratio “separation/spread”. In ICA studies, component identification is also a decision-making problem. The researcher must identify a component of interest (COI, i.e., signal) in the presence of noise (because imaging noise could be also included in the COI) from all other components containing no signal (e.g., physiological noise-related and head motion-related components, as well as components with significant voxels in white matter, cerebrospinal fluid or other brain areas of no interest). In DICI calculation, we assume that the noise in a component, as mentioned above, follows a Gaussian distribution with a fixed variance. DICI is thus, a complete characterization of the detectability of a COI. Therefore, both the hit rate (HR) and the false alarm rate (FAR) can be specifically calculated in DICI to obtain a measure of judgment that is independent of the experimenter’s criterion. As shown in Supplementary Table 1, the hit rate is also referred to as “sensitivity”, and the “1- false alarm rate” is referred to as “specificity”. DICI considers *both* sensitivity *and* specificity. The HR and the FAR are first transformed to *z* scores according to an inverted cumulative distribution function of a standard Gaussian distribution (mean = 0 and standard deviation = 1) and DICI is then obtained by subtracting *z*(FAR) from *z*(HR).

After calculating DICI values for all components and all ICA runs with different model orders, the second module of our method compares DICI values across components and different ICA runs (see Supplementary Figure 1). In this way, the component identification can take the total number of components (TNC)-induced result variability into consideration. First, for each ICA run with a specific TNC setting, all components generated by ICA are ranked in a descending order based on their DICI values. The component with the largest DICI is selected as a “candidate” for the current ICA run, which is further compared with other candidates based on all other TNC settings. The candidate component with the largest DICI value across all TNCs is chosen to be the final optimal COI. The corresponding TNC can be defined as the optimal TNC for the COI. Of note, we can also select the component with the second largest DICI value for each TNC setting; therefore, we can jointly compare components with the largest and the second largest DICI values for better component identification and to identify more than one COI. In this study, we only searched for the largest DICI values for simplification. That is, we aimed to identify one component that best represents the complete language network.

#### Automatic component identification (group-level)

To test the feasibility of our DICI algorithm, DICI was first performed on TR data at the group level (i.e., for GICA) with the template in the standard space produced in the previous step. Specifically, for each component, the HR was defined as the overlapping area between the binarized spatial map of a component and the template, divided by the total “activated” area in the template; the FAR was calculated as the area in binarized spatial map of a component but not in the template, divided by the total “inactivated” area in the template. The component with the highest DICI value across all GICA runs was labeled as the best language component at the group level. To binarize the group-level components, *t* > 1.96 (*p* < 0.05) was adopted as the threshold.

#### Automatic component identification (individual-level)

Similar to the group-level language network detection in *2.1.9*, for further demonstration of the feasibility of the DICI algorithm in individual-level language network identification, we conducted DICI at the individual level to detect the language networks for each subject in the TR group separately. Of note, the DICI-based component identification at the individual level was conducted at each individual’s own space (each subject’s native space). The language template was also individualized by warping the group-level language template (in standard space) back to each subject’s native space. To binarize the individual-level component, the threshold *z* was set in accordance with the following principles: the threshold *z* value was initially set at 1.96. However, due to heavier noise and artifacts, and fewer samples (rs-fMRI time points) at the individual level, sometimes, the *z* threshold of 1.96 is too stringent to detect any overlap with the language template. To make our DICI algorithm tolerable to extreme cases with low signal-to-noise ratio, we automatically decreased the threshold until a validated DICI value could be calculated (i.e., there were at least some overlapping voxels between individual components and the template). Specifically, this strategy was realized by: *1)* First calculating DICI based on *z* > 1.96; *2)* If, for at least one model order setting, there is at least one overlapping voxel between the thresholded component and the template, i.e., the sensitivity is larger than zero, or the specificity is less than one, conventional DICI algorithm will be conducted to select the best-fitted component; *3)* If, for all model order settings, there is no overlap after thresholding, the threshold will be continuously dropped by 0.2 until validated DICI values can be calculated, or until the threshold has dropped to the “red line” (*z* > 0.8). If the threshold of *z* > 0.8 still cannot result in an overlap, experts will visually analyze the data quality to further evaluate whether the data is useful or not. In the toolbox “PreSurgMapp” described later, we have added such a function to fully automate the DICI algorithm.

### Method validation using an independent healthy VA group

To validate the findings from the TR group, we enrolled another independent group of healthy subjects. Sixty-five healthy subjects from another research center (Center for Cognition and Brain Diseases, Hangzhou Normal University), aged between 20 and 29 years (with an average age of 20.8 years), including 39 males and 26 females, were included in this group from a local university in Hangzhou. All subjects were native Chinese speakers and right-handed. The ethics committee of the Center for Cognition and Brain Diseases in Hangzhou Normal University approved the experiment on this group of subjects. Informed consent was obtained before the experiment. The methods were carried out in accordance with the approved guidelines.

The subjects underwent a rs-fMRI scan during which they were asked to keep still, close eyes, and not to think of anything in particular. The rs-fMRI images were collected using a GE MR750 3.0 T MRI (General Electronic, Milwaukee, WI, USA) with an eight-channel coil under the following parameters: TR/TE = 2,000/30 ms, FA = 90°, slice number = 43, matrix size = 64 × 64, FOV = 220 × 220 mm, slice thickness = 3.2 mm, gap = 0, voxel size 3.4 × 3.4 × 3.2 mm^3^, dummy scan = 0, number of acquisitions = 240. The rs-fMRI scan was followed by several task fMRI scans, which are not described here as they hold no relation to the purpose of the current study. The structural MRIs were acquired using a fast spoiled gradient echo (FSPGR) sequence (TR/TE = 8100/3.1 ms, TI (preparation time) = 450 ms, matrix size = 250 × 250, FOV = 250 × 250 mm, FA = 8°, slice thickness = 1 mm, gap = 0, sagittal slices, and slice number = 176). The rs-fMRI data preprocessing and ICA procedures were same as those of the TR group. Following pre-processing, seven subjects were excluded because their head motion exceeded 2 mm in translation or 2° in rotation along any direction during the scan. 58 subjects were included in the subsequent analysis.

The language template, derived from the VA group, was used for component selection in the VA group. We used the same seed coordinates [−57, 15, 24] in BA #44 as the seed region to generate the group-level resting-state FC map for the VA group. Since the VA group did not have task-related fMRI data, this coordinate was derived from the language task activations in the TR group. We adopted this strategy based on the consideration that for future datasets that did not have task-related fMRI but only had rs-fMRI, the task activation result derived from another dataset will be used to obtain the group-level resting-state FC template for their own rs-fMRI datasets in DICI analysis.

The purpose of generating a different, data-specifc language template for the VA group was to ensure the best fit between the components and the template (since they were derived from the same dataset). However, in order to check if the template generation strategy would influence the final result, we also used the VA template (generated using the VA dataset) to identify language components from the TR subjects. We anticipated that different language network templates would still yield consistent result.

Following the identification of the group-level language network using DICI on the VA group, individual ICA was performed for ten subjects randomly selected from this group. The DICI algorithm was applied to individual-level language network identification for the VA group.

### Applying to language mapping in the TE group of glioma patients

Seven right-handed patients with gliomas in the left hemisphere, admitted into Department of Neurosurgery in Huashan Hospital, were included in the TE group. The general information of the included patients is listed in Table 1. The inclusion criteria were as follows: patients that received awake surgery and intra-operative language mapping; patients with tumors with small mass effect to achieve better registration. All patients underwent preoperative rs-fMRI scan.. The methods were carried out in accordance with the approved guidelines. The Huashan Institutional Review Board approved all experimental protocols.

**Table 1:** Demographic and clinical information of the seven glioma patients.

Patient ID	Sex	Age (y)	Tumor location	Tumor volume (cm^3^)	Pathology
1	M	23	Left frontal	49.7	Oligodendroglioma (WHO II)
2	F	48	Left frontal	5.3	Astrocytoma (WHO II)
3	M	26	Left insular	46.8	Glioblastoma (WHO IV)
4	M	39	Left insular	53.4	Oligodendroglioma (WHO II)
5	M	31	Left frontal	10.5	Astrocytoma (WHO II)
6	F	44	Left insular	53.4	Astrocytoma (WHO II)
7	M	32	Left frontal	40.4	Astrocytoma (WHO II)

The rs-fMRI was performed using an EPI sequence under the following settings: TR/TE = 2,000/35 ms, FA = 90°, slice number = 33, matrix size = 64 × 64, FOV = 210 × 210 mm, slice thickness = 4 mm, gap = 0, voxel size 3.3 × 3.3 × 4 mm^3^, dummy scan = 6 s, number of acquisitions = 240. The clinical scans varied across subjects and depended on the requirements of surgery. 3D T1-weighted MPRAGE sequence (acquired through the axial plane, TR/TE = 1,900/2.93 ms, FA = 9°, slice number = 176, matrix size = 256 × 215, FOV = 250 × 219 mm, slice thickness = 1 mm, acquisition averages = 1) and/or T2-FLAIR sequence (acquired through the axial plane, TR/TE = 9,000/99 ms, TI = 2,500 ms, FA = 150°, slice number = 66, matrix size = 256 × 160, FOV = 240 × 214 mm, slice thickness = 2 mm) were used as anatomical templates for super-positioning on the functional mapping results. Before the examination, written informed consent was obtained from the patients’ family members.

Data preprocessing and individual ICA procedures for the seven tumor patients were exactly the same as above. The DICI value-based component identification was performed with the language template derived from the TR group because the TR and TE groups were scanned with the same scanner. Because the tumors were located in the left language regions, an intraoperative language mapping by direct cortical stimulation under awake anesthesia was adopted. The details about awake anesthesia procedures and stimulation parameters have been documented in our previous studies^31,32^. The positive sites during intra-operative language mapping were recorded by neuronavigation and intra-operative images. We adopted the same method^10^ to compare intra-operative stimulation mapping (ISM) with the rs-fMRI data. A site-by-site comparison between fMRI and ISM maps was applied to obtain true-positive, false-positive, true-negative, and false-negative rs-fMRI sites.

## Results

In this section, we report the automatically identified language components by the DICI approach for each individual in the TR and TE groups. For each subject in the TR group, the individual ICA mapping result was compared with task-activation maps. For the ten subjects in the VA group, the automatically selected individual language network was confirmed by two experienced neurosurgeons (JW and JL). If inconsistent visual verification results presented, a third expert (HZ, a neuroscientist) conducted further evaluation and made the final decision. For each patient in the TE group, the ICA result was validated by ISM techniques performed by the same two neurosurgeons (JW and JL).

### Language mapping in TR group

**Figure 2:**
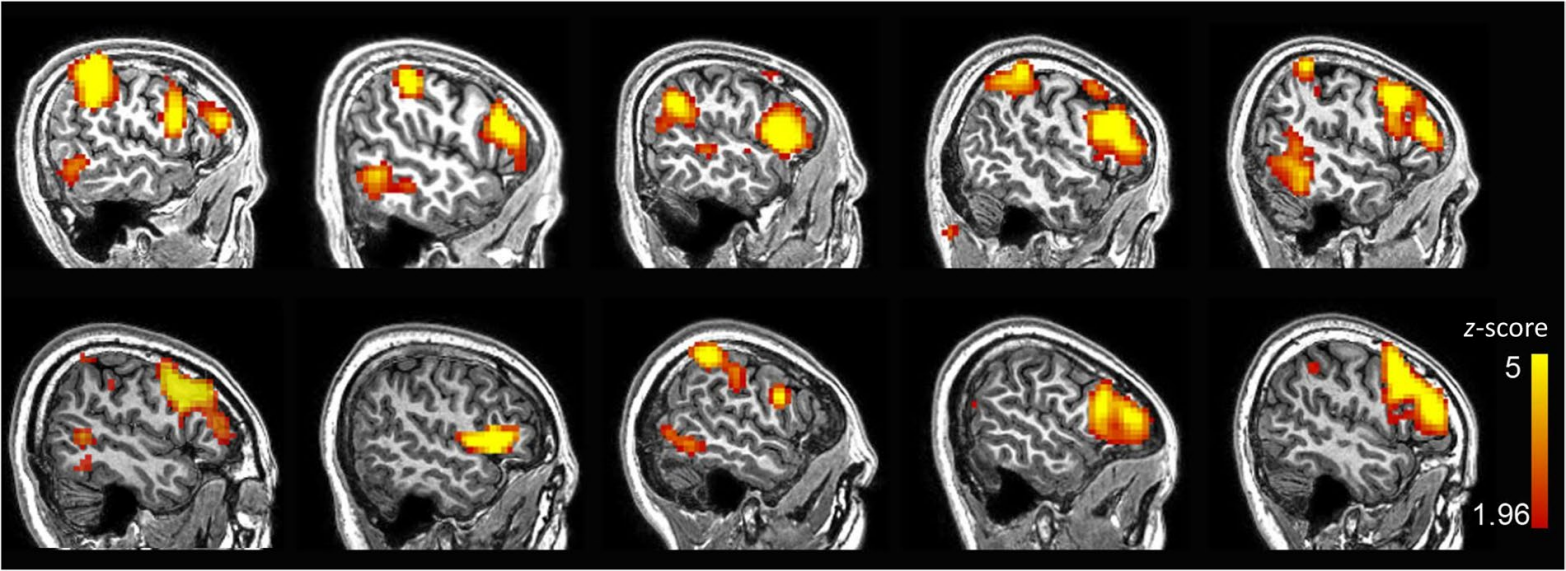
Individual language mapping result for 10 randomly selected subjects from the VA group, based on rs-fMRI and ICA. The result was z transformed and thresholded, with *z* > 1.96 (*p* < 0.05), uncorrected. The functional mapping results were superpositioned onto each individual’s T1 structural MRI. The identified components were all based on the largest DICI value across all model order cases (20, 30, 40, 50 and 60). The template used for DICI value-based automatic component identification was derived from the whole VA group.

The group-level picture naming task activation map from the TR group is presented in Fig. 1A (for a complete task activation pattern, please see Supplementary Figure 2). The peak voxel with MNI coordinates [−57, 15, 24] (see Fig. 1) was chosen as seed. Figure 1B shows the group-level FC map for the TR group by using seed correlation (for more details, please see Supplementary Table 2). The language networks were mainly distributed over the bilateral inferior/middle/superior frontal gyri, superior temporal gyrus, heads of the bilateral caudate nuclei, bilateral lentiform nuclei, and bilateral fusiform gyri (see Fig. 1 and Supplementary Figure 3). The group-level language template generated based on seed-based correlation from the rs-fMRI data showed bilateral clusters (see the second row in Fig. 1A) with both the frontal and parietal clusters having a slightly left-sided dominance. In contrast, as shown in the first row of Fig. 1A, we observe that the language task-activation map has a prominent leftward dominance. At the group level, our result is quite consistent with previous studies, where resting-state FC for the language system had usually been reported to be more symmetric as compared to language-related task activations^22^. Interestingly, in our study, individual-level ICA seemed to have produced more lateralized language-related components showing significant left lateralization for most of the subjects (Supplementary Figure 4). As shown in this result (10 subjects from the VA group, also shown in Fig. 2 for the sagittal view from the left side), 6 out of 10 subjects had prominent left-sided language resting-state FC patterns; 3 other subjects had less prominent, but still left-sided, language resting-state FC patterns; only one subject (#8) had a symmetric language FC map.

The DICI calculation at the group level demonstrated that the largest DICI value became higher when the model order was set to a larger one (red line in Supplementary Figure 6). Regardless of what the model order was, the largest DICI value could always identify language networks, and the component with the second largest DICI value was not a typical language network.

For the final group-level result, we chose the component for which the DICI value reached its maximum when the TNC was set to 60 in the TR group (Fig. 1B). This group-level ICA result largely overlapped with the group-level picture naming task activation pattern in the frontal lobe, especially on the left side (Fig. 1B).

At the individual level, the language networks in the inferior frontal gyri were identified successfully in 9 out of 10 subjects by choosing the component with the largest DICI value in all TNC settings (20, 30, 40, 50 and 60) with only one (subject #3) failing in identification. In this subject’s case, the language network corresponded to the component with the second-largest DICI value; whereas the components with the largest DICI values in all TNC settings were the motor network. We carefully compared the two largest components in this case. One explanation for the failure of detection here could be due to the cluster of language networks being small whereas the motor network is large in this case. Therefore, the language template has a larger overlap with the motor network rather than the language network.

**Figure 3:**
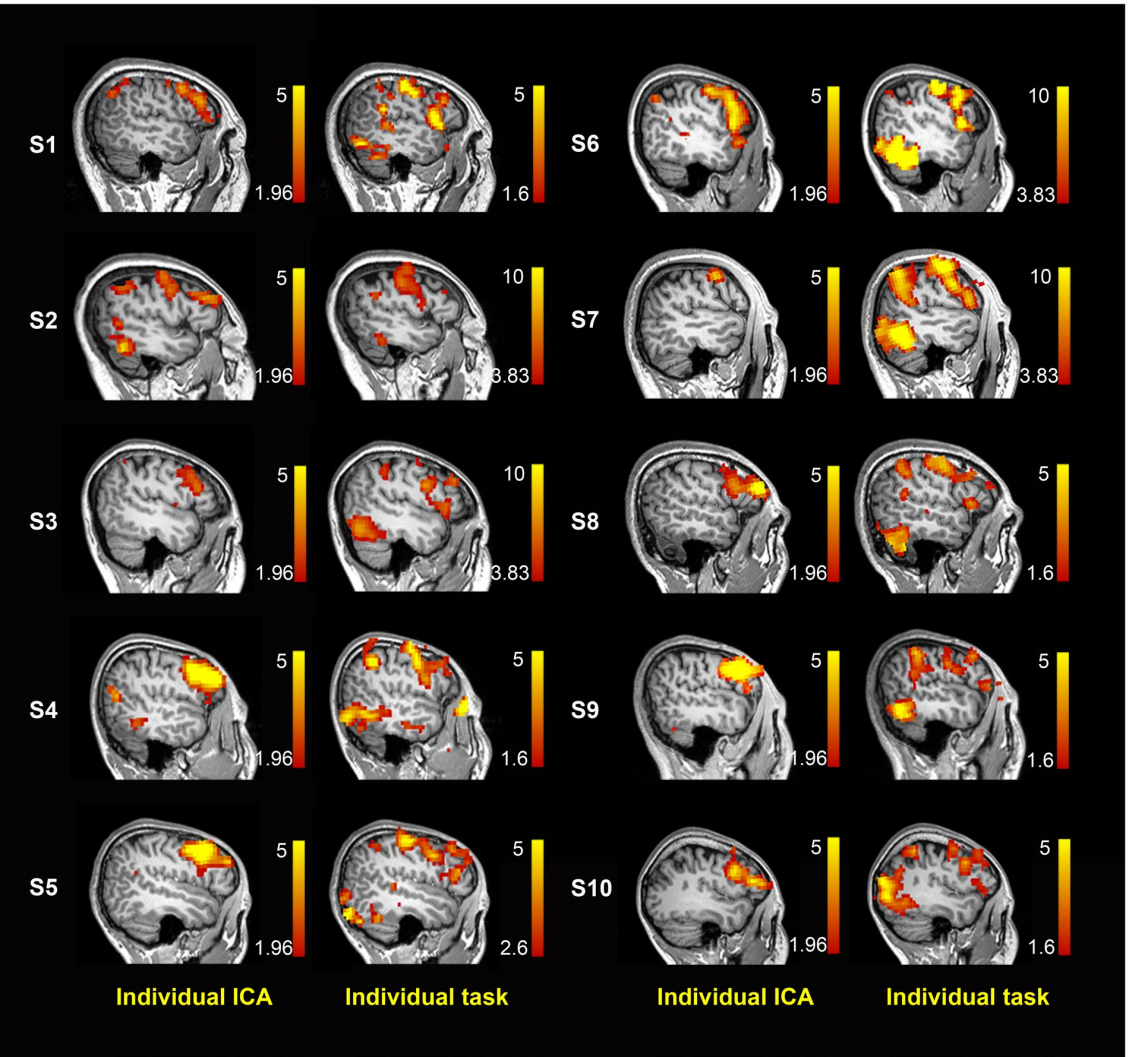
Comparison between the ICA results and the task-state results of the TR group at the individual level using the DICI value method.

As subjects in the TR group had individual picture naming task activations, they are also presented in Fig. 3 along with the ICA-derived language map for comparison. Although at the individual level, the task language mapping results overlapped with the ICA-based rs-fMRI results, especially at the left inferior frontal gyrus (Fig. 3), such an overlap was not as significant and overwhelming as that at the group level (Fig. 1B). Furthermore, the ICA-based rs-fMRI individual language mapping was more consistent across subjects, and had a more localized pattern (at the Broca’s area) than the task one (Fig. 3).

### Validation in the VA group

The group-level language FC map (VA template) applying the same seed region [−57, 15, 24] to the VA group was quite similar to, and highly overlapped with, the TR template (see Supplementary Figure 5). Our language component identification algorithm was proven to be robust. That is, the language networks of all subjects in the TR group were again successfully identified using the VA template and were highly consistent with those identified using the TR template (see Supplementary Material). For the 10 randomly selected healthy subjects in the VA group, the language networks at the individual level were all identified successfully (Fig. 2) by choosing the component with the largest DICI value in all TNC settings (20, 30, 40, 50 and 60).

### Individual-level language mapping in tumor patients (TE group)

**Figure 4:**
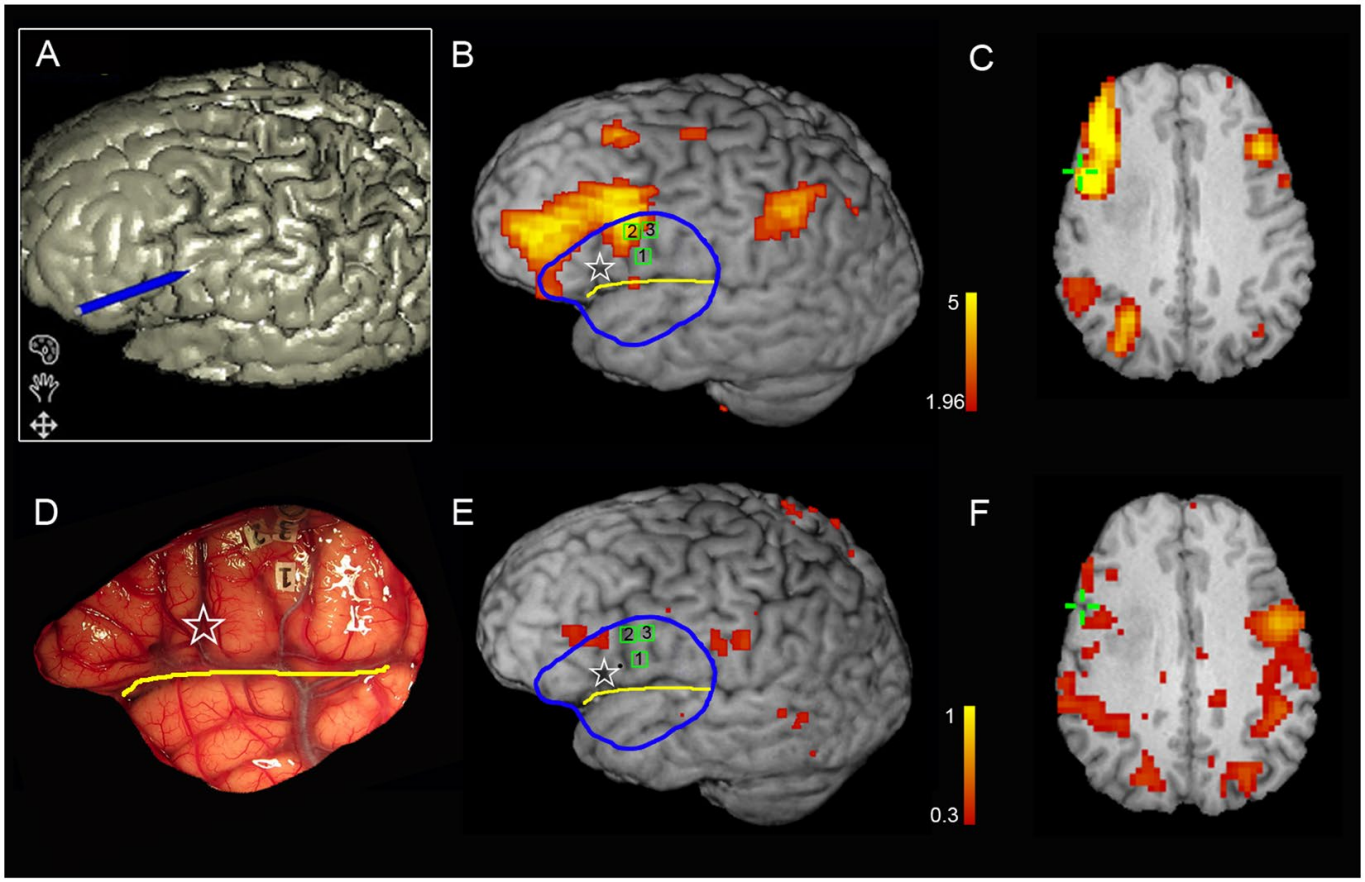
Comparison of language mapping and ISM results for the representative patient #4. The labels 1, 2 and 3 indicate the positive sites inducing speech arrest during number counting. The green cross indicates the ISM positive site of speech arrest, which was recorded using neuronavigation.

**Figure 5:**
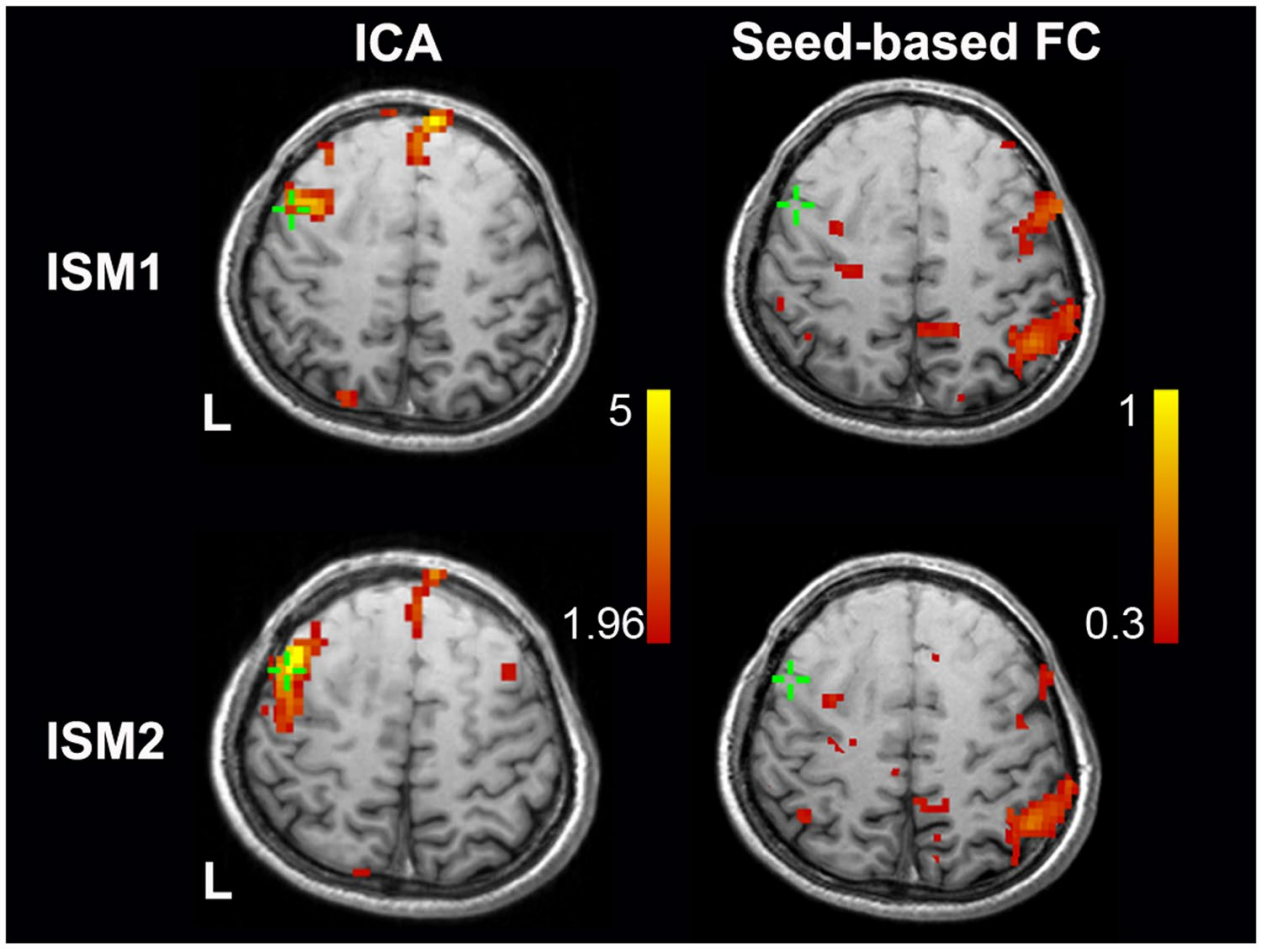
Comparison of language mapping and ISM results for the representative patient #7. The green cross indicates the ISM positive site of anomia which was recorded using neuronavigation.

In all seven patients (TE group) with glioma in the left hemisphere, the rs-fMRI and ICA-based approach successfully identified the language network in the left frontal lobe. All the identified language networks corresponded to the components with the highest DICI value in all model order cases. The comparison of the language networks obtained based on ICA and rs-fMRI with the ISM results (i.e., the gold standard) indicated that the two results were generally consistent. Specifically, of the total 23 ISM-positive sites related to language production function, 14 were located in the ICA-based rs-fMRI result (Sensitivity = 14/23, 60.9%), and 6 were located within a 1 cm radius of the ICA-based rs-fMRI result (Sensitivity = 20/23, 87.0%) (see representative cases in Figs 4 and 5, and other cases in Supplementary Figure 7). However, the seed-correlation mapping using a seed in the contralateral inferior frontal cortex with canonical coordinates showed a lower sensitivity (with 3 located in the activation (3/23, 13%), and 8 located within 1 cm (11/23, 47.8%)).

#### Representative case 1

This patient was a 39-year-old male who was admitted to the hospital due to “left insular glioma”. We performed cortical electrical stimulation (a neuronavigation screenshot is shown in Fig. 4A) for language localization and determined the location of the language area to be at the posterior inferior frontal gyrus. Based on the screenshots and the landmark sulci and gyri, three ISM positive points were found (see green squares in Fig. 4B, and also a picture labeled with Arabic numbers in Fig. 4D). Using ICA on rs-fMRI, the DICI value approached identified component #18 with TNC of 60 being the best language component (thresholded by *z* > 1.96, *p* < 0.05, uncorrected, as shown by the warm colored areas in Fig. 4B,C). The functional mapping blobs were located in Broca’s area and Wernicke’s area as well as the premotor and primary mouth motion areas. Of the three ISM positive points, two were observed in the rs-fMRI derived area, and the other was observed inferior to it within a 1-cm range. The bone window was depicted with a blue circle, within which we could see that the rs-fMRI-ICA-derived positive areas were in consistency with the ISM derived ones. However, using the same rs-fMRI data, with the seed region defined in the canonical counterpart of the Broca’s area according to the standard coordinates, the seed-based FC mapping (Fig. 4E,F) failed in the detection of positive language areas in the left hemisphere. At the three ISM positive areas, the seed-based FC values were all low (*r* = 0.002, 0.120, and 0.011).

#### Representative case 2

This patient was a 32-year-old male with glioma in the left frontal lobe. We used his preoperatively obtained rs-fMRI data to conduct ICA-based functional mapping using the DICI method. Component #26 with the TNC of 30 was determined as the language-related component (see the warm colored blobs in the left column of Fig. 5). The two ISM positive sites associated with anomia lay within the rs-fMRI and ICA-derived mapping result. In contrast, seed-based FC mapping only found activations in the parietal lobe and the dorsal part of the inferior frontal gyrus. In ISM positive regions, the seed-based FC values were low (*r* = 0.008 and 0.146).

## Discussion

### General discussion

In this study, we introduced a discriminability-index-based component identification algorithm for automatic language network extraction^33^, and investigated its feasibility, robustness and reliability for preoperative eloquent language area mapping in both healthy subjects and glioma patients. Two healthy cohorts were included, and the results were cross-validated. Individualized language network mapping could automatically be achieved without any human interference for all healthy subjects from the two groups except for one case (success rate = 95.0%). For this failed subject, the best language network identified by experts’ visual inspection corresponded to the component with the second largest DICI value; whereas the component with the largest DICI value across all model orders was less related to language function. For this case, the reason of DICI’s failure may be that the language template generated for the TR group had overlaps with the motor network in several ventral motor areas (related to the mouth and tongue movement). Thus, with this template, our algorithm tended to detect ventral motor areas as well. The second reason could be that the first and the second largest DICI values were so similar that one independent component could not cover the whole language network. That is, both of them could be regarded as the candidate components of interest but each one of them captured only part of the language network. The results showed that the rs-fMRI and ICA-derived language networks were highly consistent with the task activation results in the dominant hemisphere. In the clinical cohort consisting of 7 glioma patients, our automatically detected language areas were in concordance with the intraoperative stimulus mapping-derived gold standard (sensitivity = 87.0% while extending to a radius of 1 cm from ICA results). In contrast, the conventional seed-based correlation using “standard” coordinates achieved a sensitivity rate of only 47.8%. Such an improvement could be due to the noise reduction ability of ICA.

In recent years, rs-fMRI has greatly advanced and the basic research achievements in spontaneous brain activity and FC have been gradually applied to clinical studies of various diseases of the nervous system^14,17,34,35^. Among these applications, the implementation of rs-fMRI-based functional localization for pre-surgical planning is one of the newest and the most clinically relevant^8,22,24,36–39^. In our previous study, we conducted preliminary applications of seed-based rs-fMRI in locating eloquent language areas preoperatively^21^. Although a higher sensitivity (47.8%) as compared to the task fMRI result (35.1%) was reported, it was still not accurate enough for reliable clinical application. In the current study, we adopted a data-driven, multivariate FC analysis method to decompose rs-fMRI data, which was aimed at improving the rs-fMRI-based language mapping result. The sensitivity increased to 60.9% (87.0% when extending to a radius of 1 cm from the ICA results) and this significant improvement indicates a promising future for rs-fMRI-based pre-surgical mapping.

Although high sensitivity is reported, our approach is not yet adequate to replace the existing gold standard procedures such as ISM, considering the possible mistakes made by such an automated component identification algorithm. The ultimate goal of pre-surgical mapping is to reduce the probability of misjudgment of critical areas in surgery. Any means that can achieve this goal should be implemented. For example, based on the automatically suggested components, experts’ re-checking is of great importance. The fully automatic algorithm presented in this study is by no means a replacement of such a visual confirmation by experts, nor a replacement of the ISM technique. In addition, task activation-based pre-surgical mapping, as a traditionally well-adopted technique, should not be discarded. The rs-fMRI FC-based mapping result can be used as a good supplement in some difficult cases where gold standard procedures cannot be applied^10,11^, particularly for patients with language deficits prior to the surgery, or those with significant cognitive impairments who cannot perform complex language tasks. Another potential promising clinical usage of the DICI algorithm is that, based on the automated algorithm, multiple ICA runs with different TNC settings can be conducted and the most likely language-related components can be identified (e.g., the components with the first two largest DICI values in all TNC settings), which will dramatically reduce human labor and potential error in visual identification.

The novelty of the usage of the DICI-based post hoc analyses for individual ICA should be emphasized. Before this study, no previous studies have used a fully automated component identification method to locate the language areas individually. Visual identification^8,36^ and multiple experts’ agreement-based identification^22^ are too subjective and laborious in clinical application, and that is why ICA-based pre-surgical mapping is still not extensively used. Although Tie *et al*.^22^ attempted to adopt a goodness-of-fit (GOF) algorithm, which has been widely adopted in group ICA studies, this method has produced several “candidate” language components that necessitate further human-based identification. Our DICI approach produced only one best-fitted component, which was consistent with visual identification in 95% of the healthy subjects (19/20) and in all patients (7/7). Only in one healthy subject did DICI fail in the detection of language areas in the best-fitted component, but it was still able to detect them in the second best-fitted component. In contrast, our previous work showed that the GOF-based component identification could lead to bias and misidentification in working memory network detection (not shown). A detailed comparison of the DICI and GOF algorithms will be discussed later.

### Complexity of language area mapping

Locating eloquent language areas is more complex than defining motor areas. Studies concerning the application of rs-MRI in neurosurgery have been initiated since 2009, but most papers have reported sensorimotor mapping^8,36,38,40^. However, for language mapping, several concerns must be addressed, as elaborated below.

First, task fMRI-based language mapping has a dominant hemisphere, while rs-fMRI-derived language network is usually bilaterally distributed. The interpretation of the motor network becomes fairly straightforward by performing the “task-rest overlap” analysis^38,41^. However, in studies of language networks, a unanimous definition has never been made and language networks could easily be misidentified as frontal, frontal temporal, or frontal parietal networks. Not as consistent and reliable as the well-known default-mode network, some rs-fMRI-ICA literature did not even report language networks^42^. In this study, we also observed such a “task-rest” discrepancy in the spatial pattern. Therefore, directly using task language activation as a template to identify resting-state language network could have been sub-optimal. We used the task-related peak voxel as a seed (defined by picture naming task activation), and then generated a language template using seed-based correlation on rs-fMRI data, with which a reliable language network could be defined for subsequent DICI calculation^20,43^. We also compared our language template to the language task activation meta-analysis result (generated by searching the keyword “language” on “Neurosynth” website). Neurosynth (http://neurosynth.org) is a platform for large-scale, automated synthesis of fMRI data. It uses more than 400,000 activations reported in more than 10,000 studies, which are suitable for meta-analysis. Specifically, we used the keyword “language” and found 885 studies with 35,041 activations, based on which a language task activation “meta-analysis” map was generated (Supplementary Figure 8). As shown in the figure, our task-activation and resting-state FC results for the language network had an intersecting area in the left inferior frontal lobe (Supplementary Figure 8A), which is similar to the language task activation meta-analysis result (Supplementary Figure 8B); in the lower slice, we found four mirrored clusters from the language resting-state FC map (Supplementary Figure 8C) in the bilateral frontal and parietal regions (i.e., Broca’s area and Wernicke’s area as well as their counterparts), whereas the meta-analysis result only shows left-sided activations (Supplementary Figure 8D) with quite similar locations to our result. Our seed definition for template generation was also validated by using two independent cohorts and we found a highly overlapping group-level language connectivity pattern (see Supplementary Figure 5). Interestingly, at the individual level, task activations were more scattered and “noisy” than the rs-fMRI-ICA-derived language mapping result (see Fig. 2), although the former were significantly overlapping at the frontal region. In future, task and resting-state fMRI-based language mapping should be further compared with ISM to systematically evaluate the feasibility of the two modalities.

Secondly, language processing involves many systems, including phonologic, morphologic, and semantic systems ranging from speech perception to production^44–47^. Experimental design for language mapping using task fMRI is complex and the results depend on the chosen language task^9–11^. This will result in significant variability across research centers and studies using different language task protocols^9^. In a recent study evaluating the accuracy of task fMRI compared with ISM^10^, the sensitivity of 37.1% greatly limited its use in clinic. Resting state is a natural experimental design that facilitates result comparison and integration across centers, thus making it more suitable for pre-surgical language mapping. The “data-driven” characteristic of ICA provides unique advantages in neurosurgical functional localization, particularly, in the complex language network localization. Based on our previous experience and the reports from literature^22^, there are two components belonging to typical language networks: one is a “frontal sub-network” and the other is “temporal sub-network”. In this study, we intended to use DICI to identify the “complete” language network including both sub-networks. To do this, DICI would find an optimized ICA model order (i.e., TNC setting) to generate a single language network. This will significantly reduce the complexity of language mapping since only one best-matched language component will be identified. We must admit that since the seed region was located in the frontal lobe, and, the language template we used was also frontally dominant, the obtained ICA results could be frontally dominant. However, since language area localization in the frontal lobe is the most pivotal (as it is related to language production, an ability that is important to the quality of life) in clinical practice, our method is adequate for clinical usage as it can successfully recognize the “frontal component” of language networks. As a result, we found nearly all subjects (except subject #8, who had an equally significant parietal subdivision selected) to have a dominant frontal subdivision of the language network identified by ICA and DICI-based component selection method. The direct cause of the more frontally distributed language networks is that we selected the “language template” based on the resting-state FC analysis using the seed region defined by the picture naming task. During the picture naming task, the language production-related area, i.e., Broca’s area, was more strongly activated (see Fig. 1A, the first row). Seed-based correlation according to the left frontal language subdivision will more likely generate a connectivity map with larger and more extensive frontal area involved. Thus, when using such a template for individualized language network identification, the component with more frontal area involvement could more likely be selected by template matching. We have noticed that in previous studies^22^, the researchers separately used the frontal and the parietal parts (also, a joint map including both parts) as templates to identify the language network. When compared with expert-selected language components, the joint map-based template, and the frontal subdivision-only template resulted in better solutions^22^. This again indicates that the frontal subdivision of the language network could be more important for identifying language-related components using template-matching algorithm, and this is further validated by our study. Another consideration for future template selection is that if one needs to identify language production areas for surgical mapping, the current strategy can be used to generate a template with more frontal areas involved. If the goal is to identify language comprehension-related components, another template should be generated, e.g., a template based on the seed region placed in the temporal parietal area (Wernicke’s area), or on language interpretation-based task-related experimental design (e.g., wording listening, phonological/rhyming, and noun-verb semantic association task.). Therefore, we suggest that in the future, different templates should be prepared for different purposes, depending on the location of the tumor in the language network^48^. From the methodological viewpoint, with our DICI algorithm, we can detect multiple components (i.e., several highly ranking components) according to the two largest DICI values, higher model orders (80, 90, or more), and using templates with both frontal and parietal subdivisions included. In this way, the usage of a single template only can also achieve the detection of multiple language-related components.

Thirdly, because of the complexity and significant individual variability of language networks, and due to the absence of anatomical landmarks (unlike the “hand knot” for the motor area), direct seed-based language mapping could be problematic. In addition, lesions tend to cause displacement and functional plasticity of language areas^49–51^. This explained why our previous attempt for seed-based (using standard Broca’s counterpart coordinates as seed) language mapping only achieved 47.8% sensitivity. Although in one case (patient #1), seed-based language mapping generated a result that highly resembled the ICA-based result, our representative case report showed that the seed correlation resulted in either scattered FC pattern, or failure in detecting eloquent language areas. Therefore, we think that seed-based FC might be more suitable for motor area mapping, but it still remains questionable in language mapping. Without the requirement of *a priori* seed information, ICA thus acts as a suitable technique, especially when the artifact or noise problem is serious. However, in patients with serious brain distortion or significant language functional plasticity, the usage of ICA should be cautious. This is because ICA result requires further interpretation for COI identification, and template matching is the commonly adopted way to “interpret” the ICA result. Since our template in this study was generated using a healthy cohort, if the language areas were far away from their “normal” location, the DICI algorithm might not be able to identify language networks accurately. If the brain is much distorted by tumor mass effect, the registration error could lead to inaccuracy of the individualized template for DICI, which may lead to error in DICI. This, however, is a general problem for all functional localization algorithms using rs-fMRI and template matching. In addition to using more sophisticated registration algorithms^52,53^ that can take brain distortion and large lesions into account, based on our best clinical application experience, we would rather recommend using seed-based correlation based on the seed region defined from the unaffected side, or from the other unaffected area that has been demonstrated to be strongly functionally connected to the affected area (e.g., the Wernicke’s area to the Broca’s area). In this way, the FC map can be directly developed based on the unregistered data in the native space. A task-based fMRI session can also be of great help. In practice, we have developed a fast and easy-to-use toolbox, namely PreSurgMapp, for such type of native-space-based seed correlation analysis which can let users define the seed region in an interactive way (i.e., by a simple click, one seed region can be selected based on the T1-weighted image of the subject; doing so multiple times defines multiple seed regions for subsequent rounds of seed correlation analysis). The users can then compare all seed-correlation results and choose the best one as the language template for DICI. This software is a MATLAB-based toolbox, which is publicly available on a source code management website, GitHub (https://github.com/missy139/PreSurgMapp), as well as in the rs-fMRI forum (http://restfmri.net/forum/node/2382). The detailed manual of this toolbox can be referred to in our previous paper^48^.

### Comparisons with the traditional component identification method

In our first attempt to identify language component, we had considered achieving automatic component identification via GOF. However, the performance of GOF was not as good as we imagined (see Table 2). We further tried using GOF to identify other functional networks, such as sensorimotor and executive control networks, but the results were still not comparable to those obtained by using our algorithm (i.e., DICI). Based on Table 2, we found that the best-fitted language-related components based on the same data, and the same language template, as suggested by the GOF algorithm, were in agreement with those of the DICI algorithm for only 50% (5/10) subjects. As far as the optimal model orders suggested by the two algorithms are concerned, there was no systematic difference (i.e., GOF did not tend to suggest a smaller or larger model order). The components identified by the two algorithms were further visually compared in Supplementary Figure 9 for the five subjects with inconsistent findings. As shown in the figure, we found that for all the five subjects, DICI performed better than GOF, leading to much cleaner and more reasonable language networks. For example, for subjects #1 and #6, there were significant false positives in the parietal or occipital lobes (as indicated by the red arrows).

**Table 2:** Comparison of performance between DICI and GOF algorithms.

Subject ID	DICI		Subject ID	GOF	
	Optimal model order	Best IC #		Optimal model order	Best IC #
1	50	18	1	20*	18*
2	30	15	2	50*	35*
3	60	34	3	60	34
4	50	21	4	50	21
5	40	20	5	50*	43*
6	60	16	6	30*	13*
7	60	44	7	60	44
8	40	37	8	30*	11*
9	50	40	9	50	40
10	50	46	10	50	46

DICI: discriminability-index-based component identification algorithm; GOF: Goodness of Fit-based component identification algorithm. The comparisons were based on the subjects in the validation (VA) group. The optimal model order is determined based on DICI and GOF separately for each subject. Best IC #is the index of the best-fitted component identified. Model orders with ^*^indicate that the two algorithms produced different results.

The fundamental reason for such a different performance between the two component identification algorithms is that, even though both algorithms are template matching in nature, they have a fundamental difference in definition. GOF subtracts the sum of (or the averaged) component’s *z*-value outside of the template from the sum of (or the averaged) its *z*-value within the template; whereas DICI is calculated based on the signal detection theory from theoretical analysis of information with more meaningful definitions (to what extent the signal can be separated from the noise, calculated by the standardized hit rate subtracting the standardized false alarm rate). First, when calculating hit rate and false alarm rate, the component’s spatial map needs to be binarized, thus alleviating the possible negative effect caused by extreme values (which often exist in the ICA-derived component’s spatial maps). Secondly, GOF works on the raw *z*-values in the spatial maps; the *z*-value was calculated in an oversimplified way: for each voxel, subtracting the global mean and dividing by the standard deviation. Since ICA does not scale each component consistently (i.e., different components have different global means and standard deviations), the *z*-values across different components may not be easily comparable. However, DICI uses a reasonable distribution-based standardization and is more suitable to be used for a cross-component comparison. Thirdly, the GOF algorithm simply combines in-template *z*-scores and out-of-template *z*-scores with equal weight, but DICI balances well the sensitivity and specificity through a reasonable way (d-prime has a clear physical meaning, i.e., the distance between signal and noise distributions).

### Automatic or visual inspection-based component identification

It must be clarified that human interpretation and confirmation of the language components detected may still be required after automatic selection as another “traditional component identification method”, particularly in situations of pre-surgical evaluation, and at least certain expert input should be employed to inspect the components with high to highest d’ values. For any automatic component identification method (including DICI and other algorithms such as GOF), visual inspection is always required, especially when the result is to be used for pre-surgical planning which requires high accuracy. On the other hand, the role of automatic component identification should not be overlooked due to the following reasons.

First, for pre-surgical mapping, one may need to identify COI from the ICA results, rather than just separating noise-related components from biologically meaningful components. To achieve this, individual-level ICA decomposition must be conducted and individual-level components must be compared (either explicitly by a template-matching algorithm or inherently, by experts’ visual checking) to find out the best COI. Even an expert could sometimes make mistakes after laborious component checking. The probability of making a mistake could be increased when conducting ICA with multiple TNC settings since this parameter can significantly influence ICA result. Automatically suggested component(s) can serve as a reference for the expert to make a decision and reduce subjective errors.

Second, after obtaining ICA decomposition results, an automatic component identification algorithm can help perform the first round of component screening to remove the most irrelevant components, which can narrow down components and thus reduce human labor. After a rough screening by only selecting components with, for example, the first five largest DICI values, for the next round of visual screening by experts, the component selection process can be more accurate and time-saving. This paper is just a demonstration of the ability of DICI-based component identification. We do not recommend selecting only one component in such a highly demanding clinical application. In future, a simple modification of the DICI algorithm can be selecting the first five components as candidates and seeking expert input.

Third, in our study, we have conducted visual inspections on all components to further validate the performance of our algorithm. For all subjects, all of their components were visually evaluated by two experts and compared with the automatically chosen ones. In the event of inconsistent visual identification, a third expert helped to decide on the final result. Since the accuracy of the DICI-based language component identification is 95%, we can make a preliminary conclusion that the automatic component identification is highly accurate.

Finally, another novelty is in the performance of language network identification, where the DICI algorithm also considered different model orders’ influence on the ICA result. Rather than trying to determine an optimized model order for ICA before the decompositions, DICI did this in a more intuitive and straightforward way: going through a set of model order settings and performing ICA separately, and choosing the “best” result from the “best” results for different model orders. From our experience, this result-based algorithm was demonstrated to be better than the information theory-based algorithm such as that of Li *et al*.^54^ Our result further demonstrated that for language mapping, the model order has little effect on individual ICA. The language network got consistent when the model order was set to be higher. For individual ICA, the model order of 60 was recommended to produce better language mapping results.

### Choosing optimal ICA model order setting

We believe that there is no such relationship between the total number of rs-fMRI time points (frames) and the optimal component number (ICA model order) for identifying a particular component. Whether or not there is such an optimal component number remains an open question (see discussions below). We found from previous studies that such an empirical suggestion has been provided as a recommended setting for the total component number in ICA, such as that in Greicius *et al*.^55^, where the chosen component number is “approximately one-fourth to one-fifth the number of time points in the respective scans”. Regarding this recommendation, we believe that it may have some practical value in deciding the model order without making any assumptions, however, it cannot be considered a “rule of thumb” for deciding the setting of total component number. As an alternative, we believe that “researching goal-specific total component number optimization” could be a better way. This is the aim of our study, to use the component-of-interest and template matching-based algorithm to decide on which parameter setting is best fitted for the current component of interest and for the current data. There are no universal rules on how to choose the “correct” component number. We next present an analysis of potential factors that may affect such a parameter and give suggestions on how to choose such a parameter for different purposes.

There are several factors that may affect the “optimal” TNC. First is the signal-to-noise ratio of the rs-fMRI data. The higher level of noise the data has, the larger the component number should be set, in order to cancel out the increased noise contamination. Second is the length of the rs-fMRI data. Theoretically, this is not a factor dependent on the optimal total number of components. However, in practice, the larger our sample sizes, the more robust our components will be. Therefore, one may set a relatively small TNC if the scanning duration is very long for such a benefit. Lastly, the TNC settings can depend on the primary research goal. If we aim to find out a specific component, we may choose a TNC that can best produce this component from ICA. If, however, our goal changes to generating a dozen (~10) functional networks that are mostly reproducible by several previous ICA studies, we may need to choose 20–30 as the TNC for ICA, which would be similar to the previously widely adopted settings^26^. However, if the goal is to conduct rs-fMRI based functional parcellation of the brain for the construction of a whole-brain FC network, a larger TNC must be chosen in order to make a fine parcellation, and to ensure that the graph built has adequate nodes (each node is represented by a component). In summary, there are no universal rules on how to choose the correct TNC setting, but there are several practical suggestions, as highlighted above, for choosing a suitable parameter setting.

In this paper, ICA was conducted individually on each subject’s rs-fMRI. Compared to the group ICA, the above general methodological guidelines for individual ICA were still applicable. However, the situation can become more complicated since individual rs-fMRI data are noisier and individual ICA can lead to much greater variability. In this case, decision-making should be more case-specific, i.e., different data and different subjects may require different optimal TNC settings. In our study, we explicitly ran ICA multiple times and chose the best-fitted component from all the results and this is the main motivation of this study. Although to our best knowledge there are no studies that have done this before, of note, changing model orders and checking the resultant ICA decomposition variability has been one of the commonly used strategies in previous studies^26,55^.

### Limitations and future works

The present study has various shortcomings that must be addressed in the future. First, the threshold for ICA results is difficult to decide. In the current study, we used a flexible threshold setting strategy and determined it based on training results. In the future, automatic threshold calculation should be adopted^56,57^. Second, following further analysis of our data by the DICI method with 70 and 80 components, we discovered that with such a large model order, the language network started splitting into more components, which made it even more difficult to extract meaningful language networks. The results became more complex and the increased model-order made the ICA decomposition result more difficult to interpret. Several other components such as attention and executive control networks started to split, many of which share similar locations to that of the language subdivisions (sub-networks). In these instances, the DICI values were even smaller than those with lower (60 or lower) model orders. This is explained by more component numbers dividing the language networks as frontal and temporal-parietal components. These findings suggested that our template for the DICI-based method in this study should also be altered in order to capture language network splitting for accurately selecting the best components. In the future, we need to use a larger dataset to conduct group-level ICA with different model orders (including higher model orders) with the aim of generating different language templates for more comprehensive language component selection. Third, we only selected 10 subjects to perform individual level analysis from the VA group. In the future, we shall optimize the procedure and apply it to more individual healthy subjects. Lastly, more tumor patients should be involved in the future to further validate our method. For patients, ISM can be used as gold standard, whereas for healthy subjects whose data can be easily collected, transcranial magnetic stimulation (TMS) can be used to create a gold standard for language mapping.

## Conclusion

We propose an ICA-based language localization method for automatically identifying language networks from rs-fMRI data. Our results confirmed that preoperative language localization using the DICI value-based method is feasible. The individual-level validation using electrophysiological methods demonstrated that the application of this localization approach for identifying language components in tumor patients is promising. However, further validation studies involving larger number of patients are still necessary.

## Electronic supplementary material

Supplementary Information
