# Supplementary material for: An automated method for identifying an independent component analysis-based language-related resting-state network in brain tumor subjects for surgical planning
Source: Sci Rep. 2017 Oct 23;7:13769. doi: 10.1038/s41598-017-14248-5 (PMC5653800; doi:10.1038/s41598-017-14248-5)
Supplement: Supplementary file 1 — Supplementary Information [file 41598_2017_14248_MOESM1_ESM.doc]

**Supplementary Information for**

**An automated method for identifying an independent component analysis-based language-related resting-state network in brain tumor subjects for surgical planning**

Junfeng Lu,^1#^ Han Zhang,^2#^ N. U. Farrukh Hameed, ^1^ Jie Zhang,^1^ Shiwen Yuan,^1^  Tianming Qiu,^1^ Dinggang Shen,^2*^ Jinsong Wu^1*^

^1^Department of Neurosurgery, Huashan Hospital, Fudan University, Shanghai, China

^2^Department of Radiology and Biomedical Research Imaging Center (BRIC), University of North Carolina at Chapel Hill, Chapel Hill, NC, USA

^#^Junfeng Lu and Han Zhang contributed equally to this work.

**^*^Correspondence to:**

**Jinsong Wu**

wjsongc@126.com

Department of Neurosurgery, Huashan Hospital, Fudan University,

Shanghai, 200040, China.

Tel: +86 21 52887200

Fax: +86 21 52888771

**Dinggang Shen**

dgshen@med.unc.edu

Bioinformatics Building 3117

130 Mason Farm Road, Chapel Hill, NC 27599, USA.

Tel: +1-919-843-5420


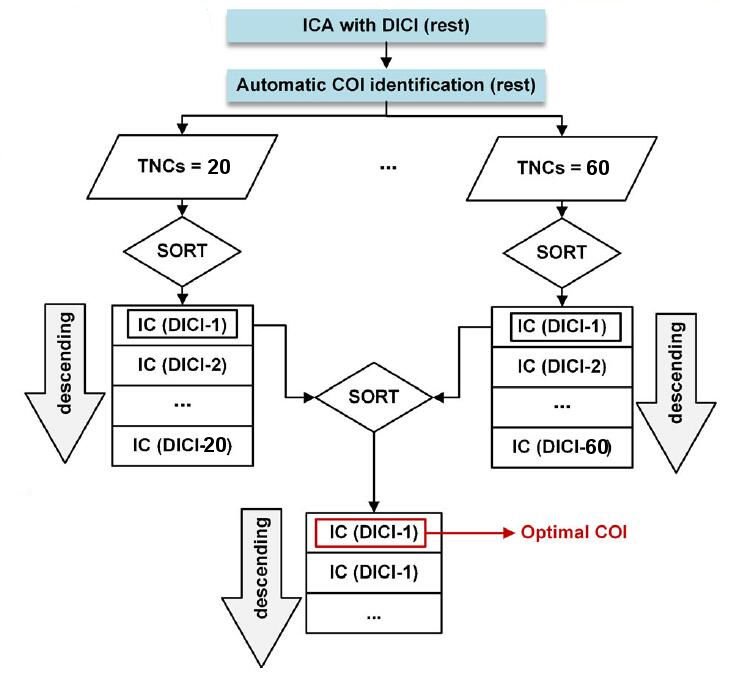


**Supplementary Figure 1.** **Flowchart of the DICI algorithm.** COI: component of interest; TNC: total number of components. Refer to supplementary table 1 below.

**Template validation**

For the 10 subjects in the TR group using different templates, 4 out of 10 subjects achieved the same result (DICI selected the same component and the same optimal ICA model order). The other subjects’ results were consistent at different ICA model orders. For instance, by using the TR template, we identified component 10 in ICA model order 40 as the language component. On the other hand, while using the VA template, this component was still the best one representing the language component in model order 40, however, the best result was chosen from those with model order 60 (based on the largest DICI value). This result indicated that our DICI value approach was neither sensitive to template choice nor to a specific dataset. Unlike the group-level ICA result where the best language mapping result was obtained with the model order 60 for the TR group and model order 20 for the VA group, individual DICI value versus “model order” plots (Supplementary Figure 5) showed that the best individualized mapping tended to correspond to higher model orders (50% of the results were with model order = 60, regardless of the source of the data or the template on which the data was based).


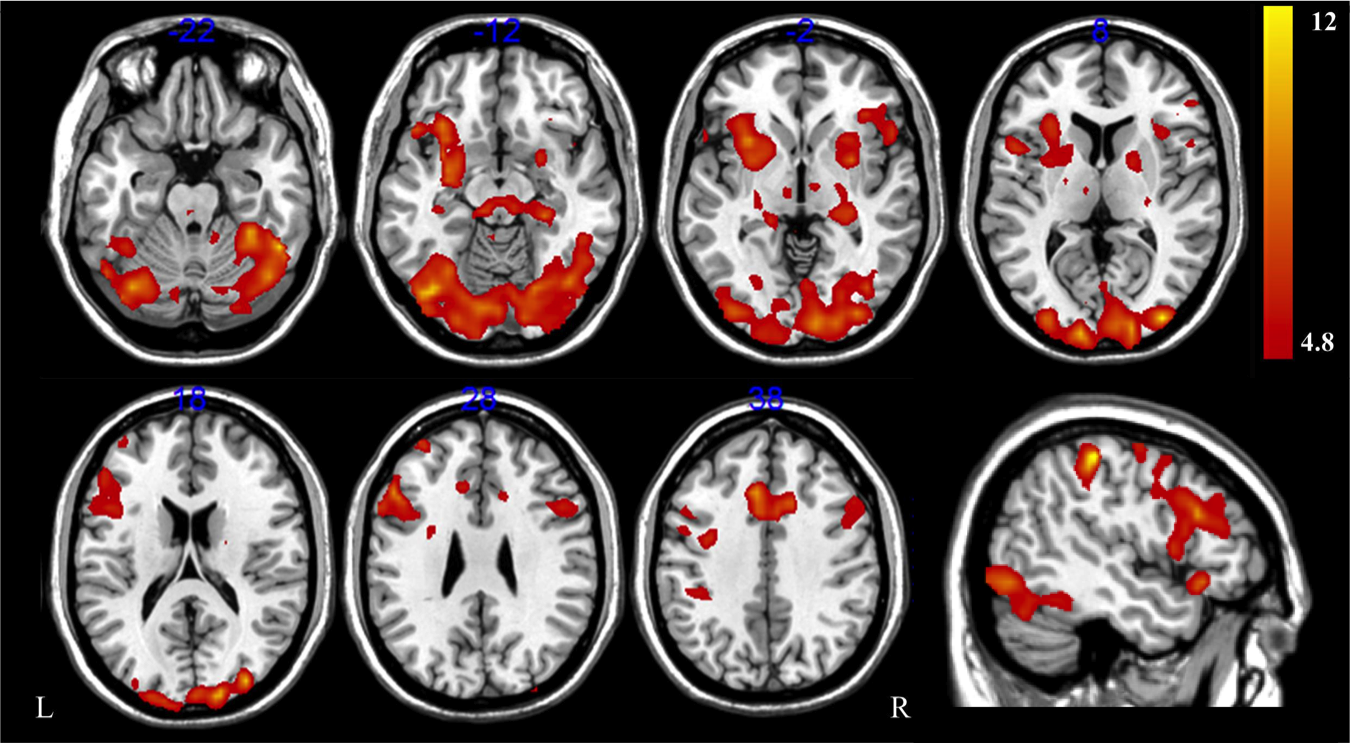


**Supplementary Figure 2.** **Complete view of the group-level picture naming task activations for the TR group.** The task activations were mainly distributed within the left inferior frontal gyrus, middle frontal gyrus, and insula; in addition, significant activations are observed in the supplementary motor area and visual cortex.


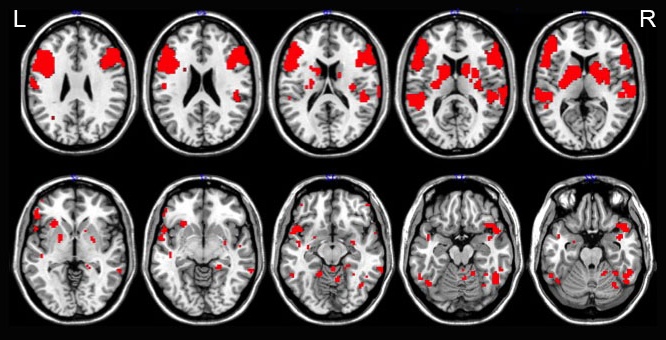


**Supplementary Figure 3. Complete view of the language template extracted from the TR group.** The peak-point coordinates located at the inferior frontal gyrus in task activation result based on language task fMRI were used as a seed point for functional connectivity analysis on the rs-fMRI data of the TR group to construct the language network template.


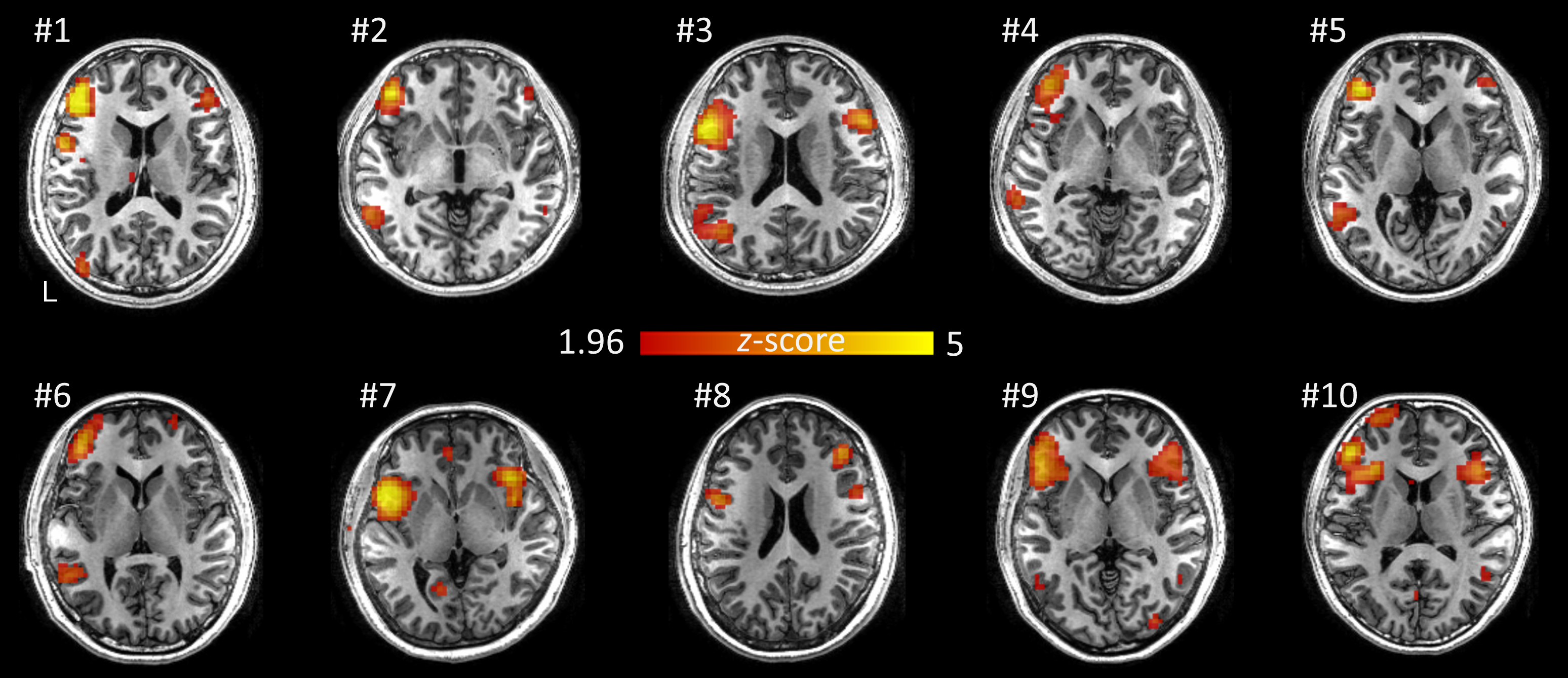


**Supplementary Figure 4. Axial view of the DICI-identified language-related components for all 10 subjects in the VA group.**


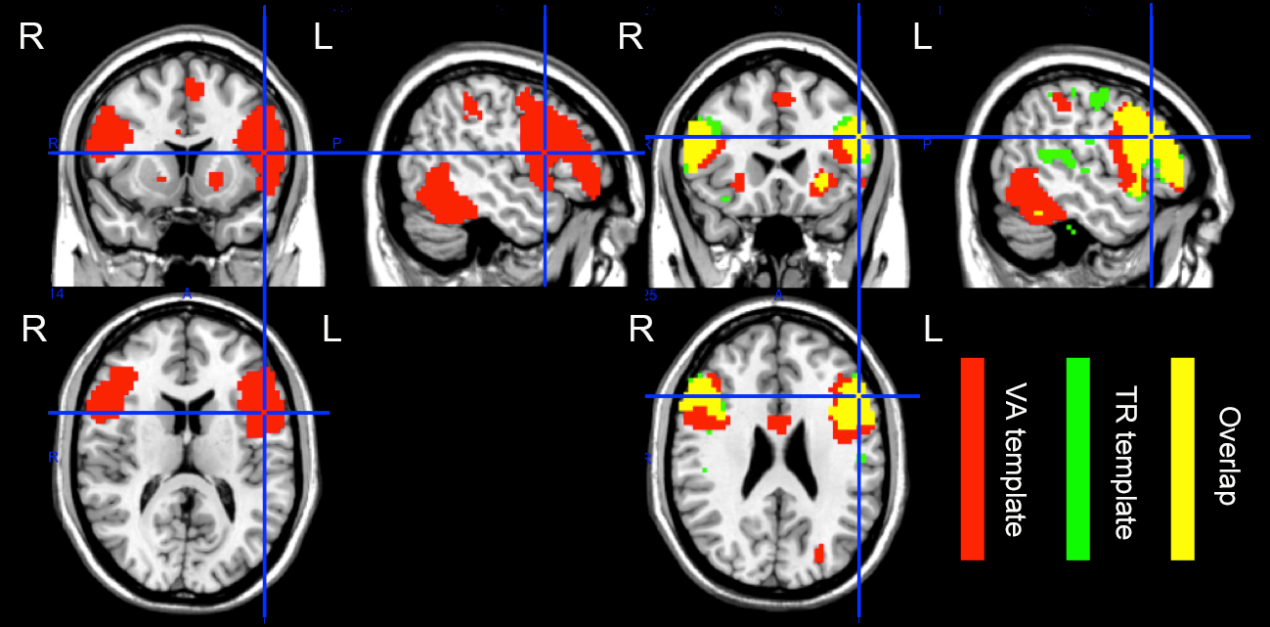


Supplementary Figure 5. The language template extracted from the VA group compared with the language template extracted from the TR group. The peak coordinate located in the inferior frontal gyrus obtained by task-state fMRI was used as a seed point for functional connectivity of the VA group and for construction of the language network map. Right: the merged image of the TR and VA template, with red indicating the VA template, green indicating the TR template, and yellow indicating the overlapping area.


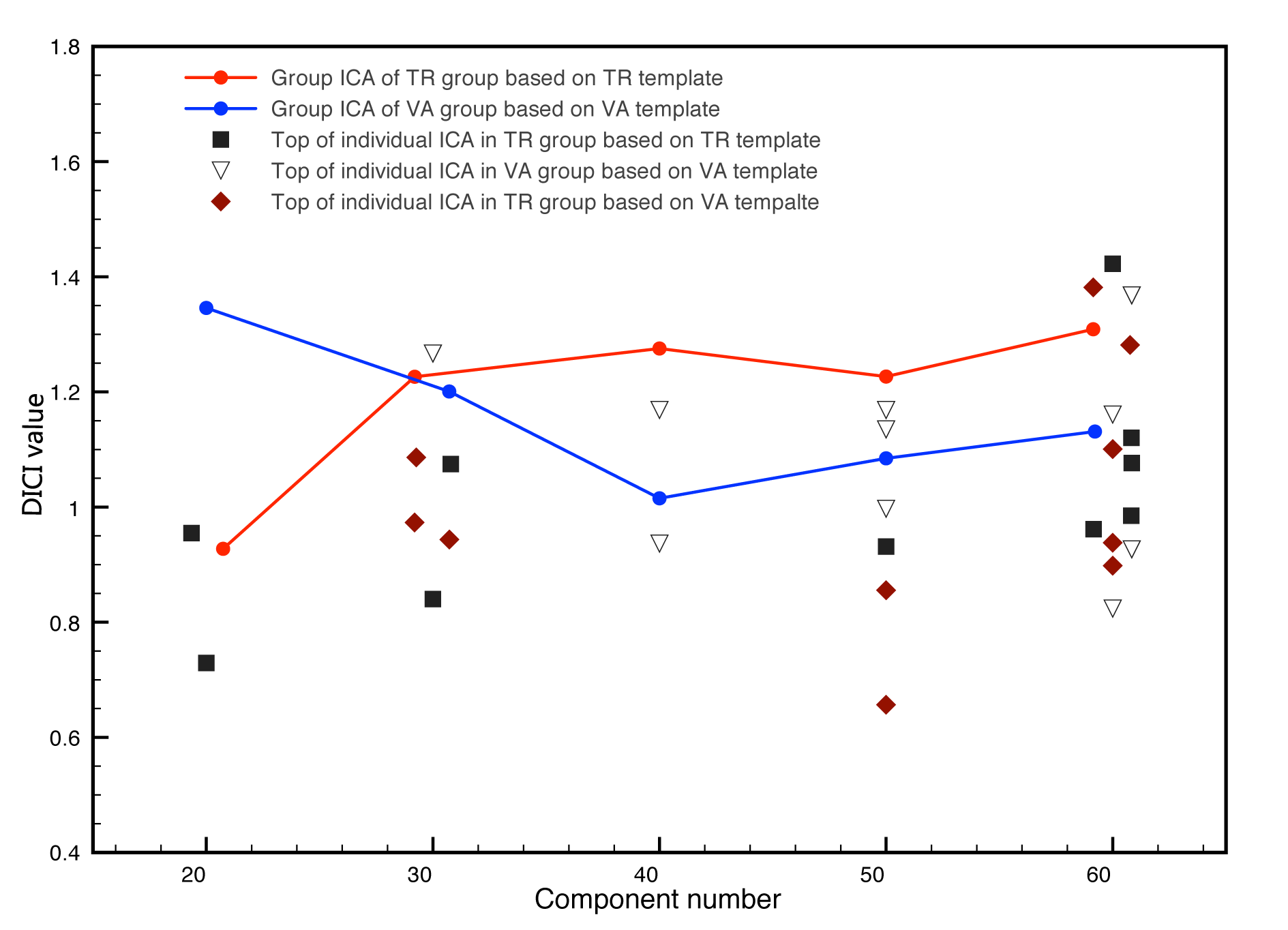


**Supplementary Figure 6. Screening results with different ICs obtained using the DICI method.** The red line indicates the highest DICI value with the corresponding ICs obtained based on the TR template of the TR group; the green line indicates the highest DICI value with the corresponding ICs obtained based on the VA template of the VA group; the other dots represent the ICs corresponding to the highest DICI value in the individual-level ICA.


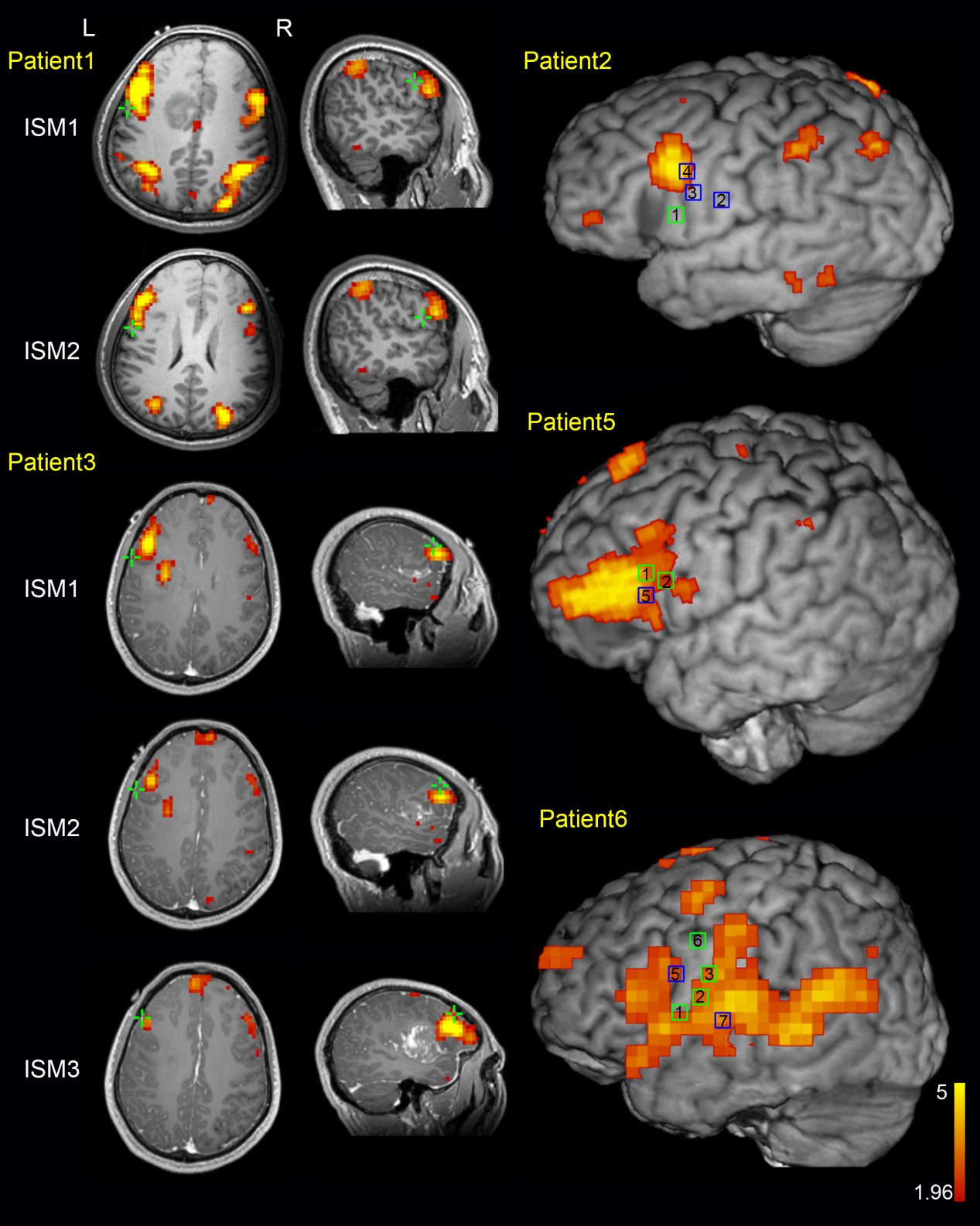


**Supplementary Figure 7. Comparison of the ICA and electrophysiological results for patients other than the two representative patients.**


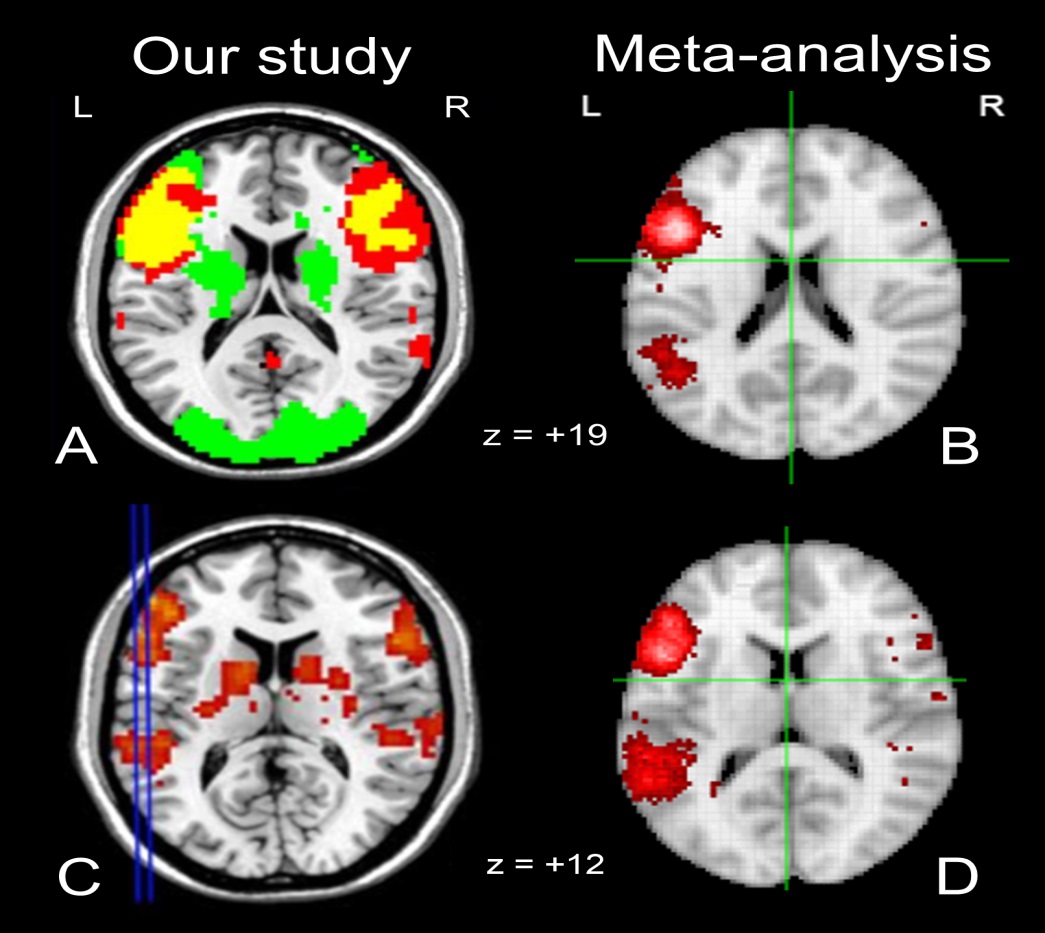


**Supplementary figure 8.** **Comparison between our resting-state functional connectivity result (A, C) and the language task-activation meta-analysis result (B, D).** Different axial slices are shown. The subplots (A, C) are same as those from **Figure 1** in the main text. Specifically, subplot A shows the overlap between task activation and resting-state functional connectivity based on our dataset (the yellow region is the overlap), and subplot B shows seed-based correlation result using the seed from task activation which has quite a symmetric pattern. For the dominant side (left side), our seed-based FC map is quite similar to the task-activation meta-analysis result.


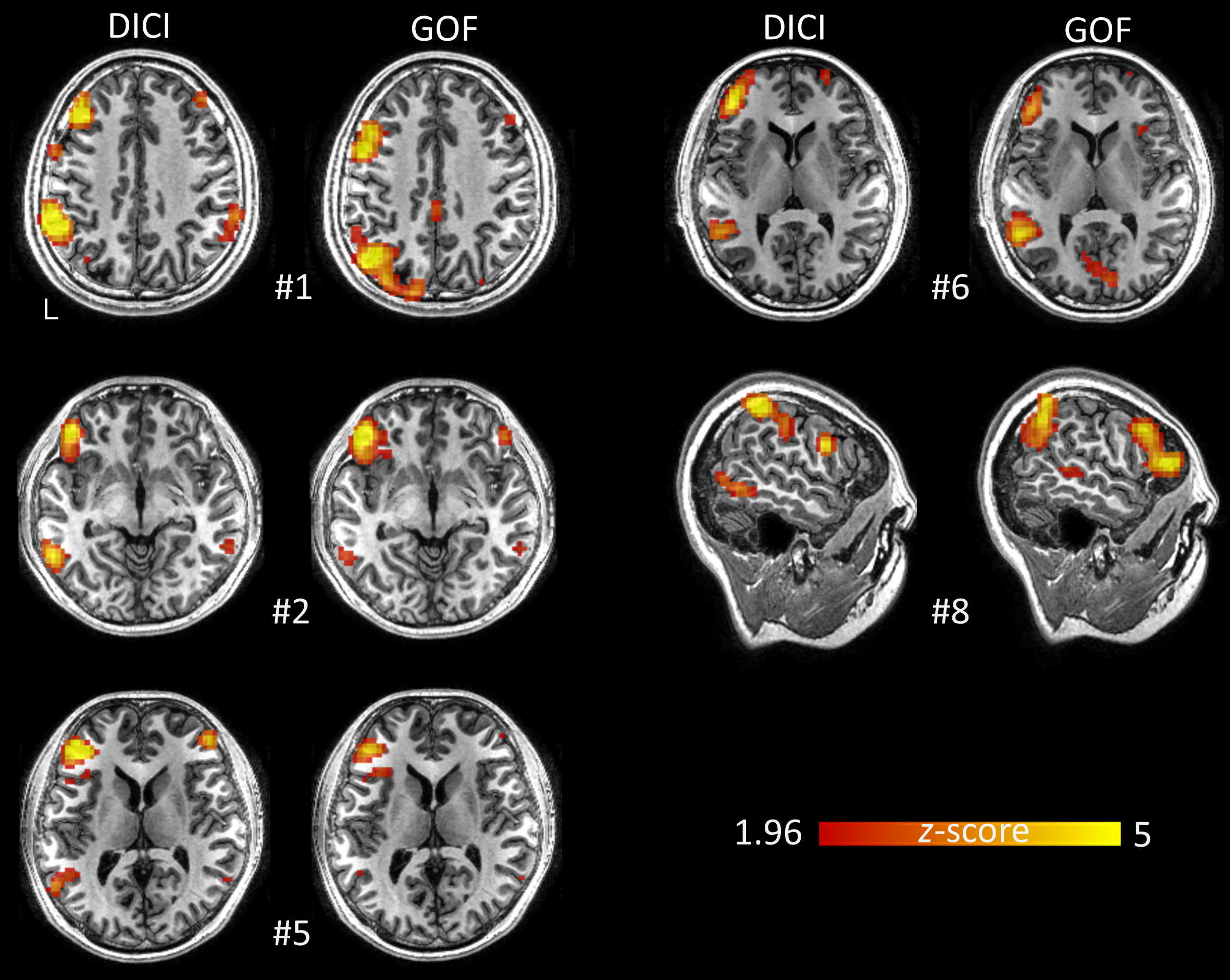


**Supplementary Figure 9. The language-related components, as suggested by both DICI and GOF, are different.** Results from the five subjects in the VA group are compared. All five subjects had different language-related component identification results based on both DICI and GOF algorithms. Red arrows indicate possible false positive and false negative identification of the language areas.

**Supplementary Table 1. Calculation of discriminability index (DICI).**

|  | Voxels labeled “1” in the component *(significant voxels)* | Voxels labeled “0” in the component *(non-significant voxels)* | **DICI = z(HR) - z(FAR)** ^a^ |
| --- | --- | --- | --- |
| Voxels labeled “1” in template *(intra-network voxels)* | **Hit**  *(true positive)* | **Miss**  *(false negative)* | **Hit rate (HR)**  = hit/ voxels labeled “1”  in template |
| Voxels labeled “0” in template *(voxels outside network)* | **False alarm** *(false positive)* | **Correct rejection**  *(true negative)* | **False alarm rate (FAR)**  = false alarm/ voxels labeled “0” in template |

a

**el: added as a corresponding author)vised the paper000000000000000000000000000000000000000000000000000000000000000000000000000**The hit rate (HR) and the false alarm rate (FAR) were transformed to *z-*scores according to an inverted cumulative distribution function of a standard Gaussian distribution (mean = 0 and standard deviation = 1).

**Supplementary Table 2. Information of the language template.**

| **L/R** | **Cluster size** | **Brain region** | **Brodmann area** | **Peak coordinates** |
| --- | --- | --- | --- | --- |
| L | 2009 | Inferior/Middle/Superior Frontal Gyrus, Precentral/Postcentral Gyrus, Superior Parietal Lobule | 6/9/40/46/4/45/44/3/38 | -57, 15, 24 |
| L | 243 | Lentiform Nucleus, Extra-Nuclear, Putamen, Caudate | - | -12, 6, 15 |
| L | 41 | Superior Temporal Gyrus | 38 | -24, 12, -27 |
| L | 32 | Inferior Frontal Gyrus, Extra-Nuclear, Insula | 13 | -27, 21, -3 |
| L | 23 | Fusiform Gyrus | 37/20 | -54, -48, -24 |
| R | 591 | Middle/Inferior/Superior Frontal Gyrus, Precentral Gyrus | 46/45/9/6/44/8 | -63, -30, 9 |
| R | 221 | Lentiform Nucleus, Extra-Nuclear, Putamen, Caudate | 13 | 30, 0, 6 |
| R | 193 | Fusiform Gyrus | 37/20 | 45, -57, -18 |
| R | 150 | Superior Temporal Gyrus, Transverse Temporal Gyrus | 41/42 | 63, -24, 9 |
| R | 108 | Superior Temporal Gyrus | 38 | 48, 15, -18 |
| R | 23 | Inferior Parietal Lobule, Supramarginal Gyrus | 40 | 45, -51, 39 |
| R | 21 | Middle Temporal Gyrus | 21 | 69, -42, -9 |

This is the group-level functional connectivity result (t-map), derived from seed-based correlation on the rs-fMRI data from the subjects in the TR group.
